# A Nonparametric Approach for Estimating the Effective Sample Size in Gaussian Approximation of Expected Value of Sample Information

**DOI:** 10.1177/0272989X251324936

**Published:** 2025-03-20

**Authors:** Linke Li, Hawre Jalal, Anna Heath

**Affiliations:** Dalla Lana School of Public Health, University of Toronto, Toronto, Canada; Child Health Evaluative Sciences, The Hospital for Sick Children, Toronto, Canada; School of Epidemiology and Public Health, University of Ottawa, Ottawa, Canada; Dalla Lana School of Public Health, University of Toronto, Toronto, Canada; Child Health Evaluative Sciences, The Hospital for Sick Children, Toronto, Canada; Department of Statistical Science, University College London, London, UK

**Keywords:** expected value of sample information, Gaussian approximation, effective sample size, health economic evaluation, value of information, uncertainty definition

## Abstract

The effective sample size (ESS) measures the informational value of a probability distribution in terms of an equivalent number of study participants. The ESS plays a crucial role in estimating the expected value of sample information (EVSI) through the Gaussian approximation approach. Despite the significance of ESS, except for a limited number of scenarios, existing ESS estimation methods within the Gaussian approximation framework are either computationally expensive or potentially inaccurate. To address these limitations, we propose a novel approach that estimates the ESS using the summary statistics of generated datasets and nonparametric regression methods. The simulation experiments suggest that the proposed method provides accurate ESS estimates at a low computational cost, making it an efficient and practical way to quantify the information contained in the probability distribution of a parameter. Overall, determining the ESS can help analysts understand the uncertainty levels in complex prior distributions in the probability analyses of decision models and perform efficient EVSI calculations.

**Highlights:**

- Effective sample size (ESS) quantifies the informational value of probability distributions, essential for calculating the expected value of sample information (EVSI) using the Gaussian approximation approach. However, current ESS estimation methods are limited by high computational demands and potential inaccuracies.
- We propose a novel ESS estimation method that uses summary statistics and nonparametric regression models to efficiently and accurately estimate ESS.
- The effectiveness and accuracy of our method are validated through simulations, demonstrating significant improvements in computational efficiency and estimation accuracy.

## Introduction

The effective sample size (ESS) quantifies the informational value of a probability distribution by representing it in terms of an equivalent number of observations contributing information to this distribution.^1^ For example, if the probability of successes p follows a beta distribution with parameters (alpha = 2, beta = 8), the ESS will be the number of successes (2) + the number of failures (8) = 10 observations. ESS can be particularly useful in computing the expected value of sample information (EVSI) using the Gaussian approximation (GA) approach.^2^ EVSI is a quantitative measure used in decision analysis to assess the potential value of acquiring additional information through a sample. This quantity represents the expected improvement in decision making or reduction in uncertainty that would result from collecting new data before making a choice or decision. In addition to its importance in understanding the information in probability distributions and for EVSI estimation, ESS also finds various applications in clinical trial designs, including prior information elicitation, sensitivity analysis, and trial protocol evaluations.^1^

Despite its usefulness, existing ESS estimation methods within the GA have limitations.^3^ This is because these methods either entail potentially high computational costs, impeding their practicality, or exhibit inaccuracies, undermining the reliability of estimates.^2^ To efficiently assess the informative level of probability distributions and simplify the EVSI calculation, we propose a novel method for estimating ESS based on summary statistics and nonparametric regression models, inspired by the nonparametric regression–based EVSI methods proposed by Strong et al.^4^ We begin by reviewing the ESS, particularly within the GA approach, and proceed to illustrate the nonparametric regression–based ESS estimation method. Subsequently, we perform a simulation to demonstrate the effectiveness and accuracy of our method, followed by a brief discussion to conclude.

## Methods

### ESS and GA

The ESS is a statistical concept that quantifies the informational value of a probability distribution through the number of hypothetical participants.^1,i^ A greater ESS signifies a more informative distribution, while a lower value indicates the opposite. The estimation of ESS serves 2 important purposes in health economic evaluation. First, it allows area experts to determine if the amount of information contained in the distribution aligns with their prior beliefs. Second, it facilitates the estimation of the EVSI using the GA approach, which is an efficient computational method that optimizes EVSI across studies with varying sample sizes.^2,5^ The efficiency of the GA method stems from the fact that ESS is computed only once to estimate EVSI across sample size.^ii^ This section introduces the concept of ESS as it is presented within the GA approach.

First, assume we have $n_{0}$ hypothetical participants, each contributing information to estimate the parameter ϕ . These participants collectively define the data collection process, where $\sigma^{2}$ represents the individual-level variance. The uncertainty in the estimate of ϕ based on this hypothetical sample size is captured by the variance $\frac{\sigma^{2}}{n_{0}}$ . Under the GA approach, the parameter ϕ is modeled as having a probability distribution p(ϕ) with mean $\mu_{0}$ and variance $\frac{\sigma^{2}}{n_{0}}$ :

(1) $$\phi \sim N\left(\mu_{0},\frac{\sigma^{2}}{n_{0}}\right),$$

where $n_{0}$ is defined as the ESS of p(ϕ) in the GA approach.

In addition, we denote the set of n observations obtained from the data collection exercise that would provide additional information on ϕ as $X_{n}$ and its sample mean as ${\bar{X}}_{n}$ . In the GA approach, it is assumed that given the parameter ϕ , ${\bar{X}}_{n}$ is approximately Gaussian distributed with mean ϕ and variance $\frac{\sigma^{2}}{n}$ :

(2) $${\bar{X}}_{n}|\phi \sim N\left(\phi ,\frac{\sigma^{2}}{n}\right).$$

Due to the conjugate nature of the distributions of ϕ and ${\bar{X}}_{n}|\phi$ , the conditional mean of ϕ given the observed data is a weighted combination of $\mu_{0}$ and ${\bar{X}}_{n}$ :

(3) $$E_{\phi }[\phi |X_{n}]=\left(1-v\right)\mu_{0}+v{\bar{X}}_{n},v=\frac{n}{n_{0}+n}.$$

Using this formulation, the ESS $n_{0}$ can be determined based on the variance of either $E_{\phi }[\phi |X_{n}]$ or ${\bar{X}}_{n}$ . This is because the marginal distributions of $E_{\phi }[\phi |X_{n}]$ and ${\bar{X}}_{n}$ are both Gaussian with variances $\frac{v\sigma^{2}}{n_{0}}$ and $\frac{\sigma^{2}}{vn_{0}}$ , respectively.^2^ As the variance of p(ϕ) is $\frac{\sigma^{2}}{n_{0}}$ , we can estimate $n_{0}$ using variance ratios. Specifically, the variance ratio between ϕ and $E_{\phi }[\phi |X_{n}]$ yields an $n_{0}$ estimation of

(4) $${\hat{n}}_{0}=n\left(\frac{{\mathrm{Var}}_{\phi }[\phi ]}{{\mathrm{Var}}_{X_{n}}\left[E_{\phi }[\phi |X_{n}]\right]}-1\right),$$

and the variance ratio between ϕ and ${\bar{X}}_{n}$ yields an $n_{0}$ estimation of

(5) $${\hat{n}}_{0}=n\left(\frac{{\mathrm{Var}}_{{\bar{X}}_{n}}[{\bar{X}}_{n}]}{{\mathrm{Var}}_{\phi }[\phi ]}-1\right).$$

Although equations 4 and 5 are derived using the Gaussian assumption, Jalal and Alarid-Escudero^2^ showed that they can be generalized to estimate $n_{0}$ for other non-Gaussian likelihood and prior combinations.^2^ For a detailed derivation of these equations, we refer the reader to their original article.^2^

### Estimate the ESS Using the Nonparametric Regression–Based Method

In this section, we review current $n_{0}$ estimation methods and note their drawbacks.^2^ We then introduce a novel approach, which uses nonparametric regression models, to efficiently and accurately estimate $n_{0}$ .

In the original GA article, Jalal and Alarid-Escudero^2^ introduced 2 methods that can be generalized to estimate $n_{0}$ for nonconjugate priors. The first method uses equation 4. For this, we need to estimate $E_{\phi }[\phi |X_{n}]$ for a large number of datasets $X_{n}$ generated from a specific data collection exercise. For each dataset, we can use Markov Chain Monte Methods (MCMC) to simulate from the posterior distribution of parameter ϕ and calculate the mean. We then compute the sample variance of these estimated posterior means to approximate ${\mathrm{Var}}_{X_{n}}\left[E_{\phi }[\phi |X_{n}]\right]$ and estimate $n_{0}$ . While this method yields accurate $n_{0}$ estimates, its reliance on repeated MCMC simulations imposes a significant computational burden, making it less practical for implementation.

The second method uses equation 5. However, since the sample mean ${\bar{X}}_{n}$ may not have the same scale as ϕ for many non-Gaussian probability distributions, equation 5 cannot be directly applied. In such cases, we need to identify other summary statistics S that share the same scale as ϕ and compare its variance with that of ϕ to estimate $n_{0}$ :

(6) $${\hat{n}}_{0}=n\left(\frac{{\mathrm{Var}}_{S}[S]}{{\mathrm{Var}}_{\phi }[\phi ]}-1\right).$$

Unlike the first method, this approach requires only summary statistics, making it computationally efficient. However, the key limitation lies in identifying suitable summary statistics S that both share the same scale as ϕ and have appropriate variability, which can be challenging in real-world case studies. For instance, when appropriate summary statistics are not readily available, this method may introduce bias into the $n_{0}$ estimation.

We propose a novel approach that substantially reduces the computational burden of estimating $E_{\phi }[\phi |X_{n}]$ required in the first method. Our method, inspired by the EVSI estimation framework proposed by Strong et al.,^4,6^ uses nonparametric regression models to estimate $E_{\phi }[\phi |X_{n}]$ directly. To achieve this, we need to first determine a low-dimensional summary statistic of the dataset $X_{n}$ , denoted $T\left(X_{n}\right)$ . Note that, different from the current summary statistics–based method, the regression-based method does not require $T\left(X_{n}\right)$ to be converted to the same scale as ϕ , which makes it simpler to specify. Ideally, $T\left(X_{n}\right)$ should efficiently capture all the information in the original dataset, with sufficient statistics being the most desirable choice.^7^ However, when sufficient statistics are not practically obtainable, alternatives such as maximum likelihood estimate (MLE) or moment estimators are often used, as they still retain most of the relevant information.^8^

After we find $T\left(X_{n}\right)$ , using the formulation suggested by Strong et al.,^4^ ϕ can be expressed as the function of $T\left(X_{n}\right)$ , plus a zero mean error term ϵ :

(7) $$\phi =g\left(T\left(X_{n}\right)\right)+\epsilon .$$

This equation suggests that the conditional expectation $E_{\phi }[\phi |X_{n}]$ can be approximated by the fitted value of a nonparametric regression model that takes ϕ as the response and $T\left(X_{n}\right)$ as the predictor.^9^ Therefore, our proposed method begins by fitting this nonparametric regression and then extracting the fitted values from this regression model. Next, ${\mathrm{Var}}_{X_{n}}\left[E_{\phi }[\phi |X_{n}]\right]$ is approximated by the variance of these fitted values. Finally, $n_{0}$ can then be estimated from equation 4.

The choice of regression models is flexible, ranging from statistical models such as splines and Gaussian process regression to machine learning approaches such as Bayesian additive regression trees and neural networks. In our case study, we used splines because these case studies contain 4 or fewer summary statistics. However, when the number of summary statistics exceeds 5, Gaussian process regression is more appropriate due to its flexibility in modeling complex relationships.^4^ The detailed algorithm for estimating $n_{0}$ using the nonparametric regression–based method is summarized in Box 1.

**Box 1:** The algorithm for estimating the effective sample size ( $n_{0}$ ) using the nonparametric regression-based method.

**Algorithm 1:** Nonparametric regression–based effective sample size estimation algorithm
1: **Generate samples of parameters and datasets:** • Generate M samples of parameter ϕ from the probabilistic distribution p(ϕ) , denoted $\phi^{1},\ldots ,\phi^{M}$ . • For m from 1 to M , use the sample $\phi^{m}$ to generate a sample of the dataset at the sample size n , $X_{n}^{m}$ , and compute its summary statistics $T\left(X_{n}^{m}\right)$ . 2: **Fit the regression model and estimate the effective sample size**: • Regress the parameter samples $\phi^{m}$ on the summary statistics $T\left(X_{n}^{m}\right)$ using a nonparametric regression model. • Extract the fitted values from the regression model and calculate their sample variance, denoted ${\hat{\mathrm{Var}}}_{X_{n}}\left[E_{\phi }[\phi |X_{n}]\right]$ . • Calculate the sample variance of $\phi^{1},\ldots ,\phi^{M}$ , denoted ${\hat{\mathrm{Var}}}_{\phi }[\phi ]$ . • Estimate the effective sample size of ϕ using $n\left(\frac{{\hat{\mathrm{Var}}}_{\phi }[\phi ]}{{\hat{\mathrm{Var}}}_{X_{n}}\left[E_{\phi }[\phi |X_{n}]\right]}-1\right)$ .

### Case Study: Examining the Accuracy of the Nonparametric Regression–Based $n_{0}$ Estimation

We assess the accuracy of the proposed method across 7 diverse data collection scenarios, including settings where ϕ is univariate and multivariate. The distributions chosen for prior and data generation, respectively, for 7 scenarios are 1) beta-binomial, 2) gamma-exponential, 3) Poisson-gamma, 4) Gaussian-Weibull, 5) Dirichlet-multinomial, 6) truncated normal-binomial, and 7) transformed beta-exponential. For each scenario, we compute $n_{0}$ using the original summary statistic and MCMC methods and our nonparametric regression–based method. Moreover, for scenarios 1 to 4, where closed-form posterior distributions exist, we derive the analytic value of $n_{0}$ and treat its value as the gold standard for comparison. For the remaining scenarios, where analytic solutions are unavailable, the $n_{0}$ value obtained from the MCMC method serves as the gold standard. Table 1 details the prior distributions, likelihood functions, and $n_{0}$ for each scenario.

**Table 1:** Probability Distribution, Likelihood Function and, Effective Sample Size ( $n_{0}$ ) Using the Analytic Results for 7 Data Collection Exercises^a^

Simulation Setting				
	Experiment 1	Experiment 2	Experiment 3	Experiment 4
Prior	P~Beta(α=4, β=6)	λ~Gamma(α=20, β=10)	λ~Gamma(α=50, β=100)	P~Dirichlet(α= (10,5,8))
Likelihood	X~Binomial(n= 20,P)	$X_{i}\sim \mathrm{Exp}\left(\lambda \right)$ i=1,…,100	$X_{i}\sim \mathrm{Possion}\left(\lambda \right)$ i=1,…,100	X~Multinomial(n= 50,P)
Analytic $n_{0}$	$n_{0}=\alpha +\beta =10$	$n_{0}=\alpha =20$	$n_{0}=\beta =100$	$n_{0}=10+5+8=23$
$n_{0}$ estimates and 95% CI				
Summary statistics method	9.97(9.78,10.18)	24.17(23.39,24.92)	100.15(98.60,101.70)	23.26(22.39,23.38)
Regression-based method	10.00(9.80,10.20)	20.92(20.23,21.58)	98.83(97.25,100.37)	23.12(22.64,23.58)
MCMC method	10.05(9.73,10.37)	22.48(21.02,24.02)	99.02(97.14,100.96)	23.98(23.16,24.80)
Computational time of $n_{0}$ (min)				
Summary statistics method	0.01	0.01	0.01	0.01
Regression-based method	0.01	0.01	0.01	0.01
MCMC method	19.72	12.62	24.91	38.17
Simulation Settings				
	Experiment 5	Experiment 6	Experiment 7	
Prior	$\theta \sim N(\mu_{0}=1,$ $\sigma^{2}=0.04)$	$P\sim \mathrm{TN}(\mu_{0}=0.2,\sigma^{2}=0.01,$ a=0,b=1) ^b^	λ=−(1−P), P~Beta(α=4,β=6) ^c^	
Likelihood	$X_{i}\sim \mathrm{Weibull}\left(v=1,\lambda =1/\theta \right)$ i=1,…,100	X~Binomial(n=20,P)	$X_{i}\sim \mathrm{Exp}\left(\lambda \right)$ i=1,…,100	
Analytic $n_{0}$	—	—	—	
$n_{0}$ estimates and 95% CI				
Summary statistics method	29.96(27.48,32.45)	17.02(16.03,18.03)	7.30(5.75,8.84)	
Regression-based method	24.92(22.66,27.14)	17.34(16.38,18.26)	4.96(3.58,6.34)	
MCMC method	25.13(22.91,27.30)	17.05(16.44,18.26)	4.94(3.58,6.23)	
Computational time of $n_{0}$ (min)				
Summary statistics method	3.45	0.01	0.01	
Regression-based method	3.52	0.01	0.01	
MCMC method	24.15	3.31	285.13	

a The $n_{0}$ estimates, their 95% confidence interval, and associated computational time are given by the summary statistics approach. The nonparametric regression–based approach and MCMC approach are also reported.

b The prior distribution for the parameter *P* follows a truncated normal distribution with a domain ranging from 0 to 1, a mean of 0.2, and a variance of 0.01.

c The prior distribution of the parameter θ is defined as the negative natural logarithm of 1 −*P*, where *P* follows a beta distribution with parameters α = 4 and β = 6.

To calculate the ESS using our nonparametric regression–based method, we generate $10^{5}$ parameter samples from each prior distribution. Each parameter sample is then inputted into the likelihood function of the study design to generate $10^{5}$ datasets. Subsequently, we compute the MLE as the summary statistic for each dataset. For our method, we then regressed the parameter samples on the MLEs using a spline. These $10^{5}$ fitted values were extracted from the regression model and used to compute $n_{0}$ according to equation 5. The summary statistics–based method also used the MLEs to estimate $n_{0}$ by equation 6, while the MCMC method was based on $10^{4}$ posterior samples for each dataset to estimate $E_{\phi }[\phi |X_{n}]$ .^2^ We also employ the bootstrap method to generate the 95% confidence interval of $n_{0}$ estimates for each method and have included the detailed procedure in the Appendix.

## Results

Table 1 provides the estimated $n_{0}$ values and the associated time to compute the ESS for each data collection process using distinct estimation methods. For the data collection exercises 1 to 4, the estimates of $n_{0}$ obtained from the nonparametric regression–based methods consistently aligned with the analytic results. In contrast, the summary statistics–based method overestimates the $n_{0}$ for the second experiment. Although the analytic result for $n_{0}$ is unavailable for the data collection exercises 5 to 7, the estimates provided by the nonparametric regression–based method as well as the 95% confidence interval remain consistent with that obtained from the MCMC method. Conversely, the summary statistics–based method may overestimate $n_{0}$ for these experiments. In terms of computational efficiency, both the nonparametric regression–based method and the summary statistics-based method can estimate $n_{0}$ within seconds or minutes, whereas the MCMC method takes about an hour in similar settings.

## Discussion

This article introduces a novel approach for efficiently quantifying the amount of information contained in a probability distribution (ESS) by combining summary statistics and nonparametric regression models. Our method provides an estimation of the ESS for various probability distributions based on a GA. While effective, this method depends on having a sufficiently large training sample for the nonparametric regression model to converge, and the choice of summary statistics is critical. Ideally, the summary statistics should capture most of the information in the original dataset, with MLE being a particularly good choice, as they are not only robust but also computationally efficient. The accuracy and efficiency of the proposed method are validated through 7 experimental data collection designs involving various types of probability distributions.

In addition to EVSI calculation, our efficient ESS estimation approach can also be used in other applications. This encompasses evidence synthesis in clinical trials, as explored by Morita et al.,^1^ and informing parameter distributions in medical decision making. For instance, our method can be particularly useful when the uncertainty distribution for a given model input is complex and ESS cannot be readily determined from the parameters of the distribution. In such instances, ESS provides a valuable metric to quantify the number of participants contributing information to a specific input. This insight can be compared with an expert’s intuition, and if there are discrepancies, the uncertainty representation can be revised accordingly.

Overall, ESS is a useful metric that can be readily used to inform EVSI and guide input distributions in medical decision making. Our proposed approach can help estimate ESS more accurately and efficiently.

## Supplemental Material

sj-pdf-2-mdm-10.1177_0272989X251324936 – Supplemental material for A Nonparametric Approach for Estimating the Effective Sample Size in Gaussian Approximation of Expected Value of Sample Information Supplemental material, sj-pdf-2-mdm-10.1177_0272989X251324936 for A Nonparametric Approach for Estimating the Effective Sample Size in Gaussian Approximation of Expected Value of Sample Information by Linke Li, Hawre Jalal and Anna Heath in Medical Decision Making

sj-pdf-3-mdm-10.1177_0272989X251324936 – Supplemental material for A Nonparametric Approach for Estimating the Effective Sample Size in Gaussian Approximation of Expected Value of Sample Information Supplemental material, sj-pdf-3-mdm-10.1177_0272989X251324936 for A Nonparametric Approach for Estimating the Effective Sample Size in Gaussian Approximation of Expected Value of Sample Information by Linke Li, Hawre Jalal and Anna Heath in Medical Decision Making

sj-rmd-1-mdm-10.1177_0272989X251324936 – Supplemental material for A Nonparametric Approach for Estimating the Effective Sample Size in Gaussian Approximation of Expected Value of Sample Information Supplemental material, sj-rmd-1-mdm-10.1177_0272989X251324936 for A Nonparametric Approach for Estimating the Effective Sample Size in Gaussian Approximation of Expected Value of Sample Information by Linke Li, Hawre Jalal and Anna Heath in Medical Decision Making

## Notes

i In Monte Carlo methods such as Markov Chain Monte Methods or importance sampling, ESS refers to the number of independent samples that would yield the same precision as the correlated or weighted samples. This definition differs from the concept of ESS as discussed in this article.

ii To estimate EVSI, ESS can be combined with the nonparametric regression models and Taylor series expansions to efficiently approximate the distribution of the conditional economic benefit and estimate EVSI. We refer readers to the article by Li et al.^5^ for a comprehensive explanation of the EVSI estimation method.
