# Supplementary material for: A Nonparametric Approach for Estimating the Effective Sample Size in Gaussian Approximation of Expected Value of Sample Information
Source: Med Decis Making. 2025 Mar 20;45(4):370–5. doi: 10.1177/0272989X251324936 (PMC11992650; doi:10.1177/0272989X251324936)
Supplement: sj-pdf-2-mdm-10.1177_0272989X251324936 – Supplemental material for A Nonparametric Approach for Estimating the Effective Sample Size in Gaussian Approximation of Expected Value of Sample Information [file sj-pdf-2-mdm-10.1177_0272989X251324936.pdf]

Appendix

Bootstrap Confidence Interval for n_0 Estimates

This section outlines the method for computing the confidence interval (CI) for nonparametric regression-based estimates of n_0.

To establish the confidence interval, we first draw M samples of the parameter ϕ from its probabilistic distribution $p(\phi)$, denoting these samples as $\phi^1, \ldots, \phi^M$. For each ϕ^m, where m ranges from 1 to M, we generate a corresponding dataset sample of size n, represented as $\boldsymbol{X}_n^m$, and compute its summary statistics $T(\boldsymbol{X}_n^m)$.

Subsequently, we perform a regression of the parameter samples ϕ^m against the summary statistics $T(\boldsymbol{X}_n^m)$ using a nonparametric regression model. The fitted values extracted from this model are represented as $\mathbb{E}_\phi[\phi|\boldsymbol{X}_n^m]$, for $m = 1, \ldots, M$. By bootstrapping M samples of the fitted values and ϕ with replacement from their respective distributions $\phi^1, \ldots, \phi^M$ and $\mathbb{E}_\phi[\phi|\boldsymbol{X}_n^1], \ldots, \mathbb{E}_\phi[\phi|\boldsymbol{X}_n^M]$, we calculate the sample variances $\widehat{\text{Var}}_\phi[\phi]$ and $\widehat{\text{Var}}_{\boldsymbol{X}_n}[\mathbb{E}_\phi[\phi|\boldsymbol{X}_n]]$. This allows us to estimate n_0 as follows:

$$n\left(\frac{\widehat{\text{Var}}_\phi[\phi]}{\widehat{\text{Var}}_{\boldsymbol{X}_n}[\mathbb{E}_\phi[\phi|\boldsymbol{X}_n]]} - 1\right)$$

Repeating this resampling process L times yields n_0 estimates $n_0^1, \ldots, n_0^L$. From these L estimates, we construct the 95% confidence interval for nonparametric regression-based n_0 as:

$$n_0 \pm z_{0.025}\sqrt{\text{Var}_{\text{boot}}}, \tag{8}$$

where Var_{boot} is calculated as:

$$\text{Var}_{\text{boot}} = \frac{1}{L}\sum_{l=1}^{L}\left[n_0^l - \frac{1}{L}\sum_{l=1}^{L}(n_0^l)\right]^2. \tag{9}$$

This bootstrap procedure is also applicable for computing confidence intervals for summary statistics and MCMC-based methods if those approaches are used to estimate n_0 in each resampling step.
