# Supplementary material for: A Nonparametric Approach for Estimating the Effective Sample Size in Gaussian Approximation of Expected Value of Sample Information
Source: Med Decis Making. 2025 Mar 20;45(4):370–5. doi: 10.1177/0272989X251324936 (PMC11992650; doi:10.1177/0272989X251324936)
Supplement: sj-pdf-3-mdm-10.1177_0272989X251324936 – Supplemental material for A Nonparametric Approach for Estimating the Effective Sample Size in Gaussian Approximation of Expected Value of Sample Information [file sj-pdf-3-mdm-10.1177_0272989X251324936.pdf]

```
require(splines2)
require(boot)

set.seed(3000615)
################## Experiment 1, Beta-Binomial ##################
### 1. Obtain samples of theta from the prior distribution
nSim <- 1e5
alpha <- 4
beta <- 6
n_0 <- alpha + beta
P_SE <- rbeta(nSim, alpha, beta)
n_trial <- 20
X_mean <- rbinom(nSim, n_trial, P_SE) / n_trial

## (1) Variation ratio approach
start_time <- Sys.time()

X_mean <- rbinom(nSim, n_trial, P_SE) / n_trial
# post_X <- n_0 / (n_0 + n_trial) * alpha / (alpha + beta) +
#   n_trial / (n_0 + n_trial) * X_mean
# n_0_hat_ratio_1 <- n_trial * (var(P_SE) / var(post_X) - 1);n_0_hat_ratio_1
n_0_hat_ratio_1 <- n_trial * (var(X_mean) / var(P_SE) - 1);n_0_hat_ratio_1
end_time <- Sys.time()
end_time - start_time

## Bootstrat CI
```

```
n_0_dataset <- cbind(P_SE,X_mean)

n_0_func_ss_med <- function(n_trial, data, indices)
{
  d <- data[indices,] # allows boot to select sample
  n_0_estimates <- n_trial * (var(d[,2]) / var(d[,1]) - 1)
  return(n_0_estimates)
}

CI_results <- boot(data=n_0_dataset, statistic=n_0_func,
          R=1000, n_trial=20)

n_0_hat_ratio_1_ci <- boot.ci(CI_results, type="norm");n_0_hat_ratio_1_ci




## (2) Nonparametric reg model approach
start_time <- Sys.time()
X_mean <- rbinom(nSim, n_trial, P_SE) / n_trial
post_X_gam <- lm(P_SE ~ bSpline(X_mean, degree = 5))

n_0_gam_1 <- n_trial * (var(P_SE) / var(post_X_gam$fitted.values) -
             1);n_0_gam_1
end_time <- Sys.time()
```

```
end_time - start_time


## Bootstrat CI

n_0_dataset <- cbind(P_SE,post_X_gam$fitted.values)


n_0_func <- function(n_trial, data, indices)

{

  d <- data[indices,] # allows boot to select sample

  n_0_estimates <- n_trial * (var(d[,1]) / var(d[,2]) - 1)

  return(n_0_estimates)

}




CI_results <- boot(data=n_0_dataset, statistic=n_0_func,

                R=1000, n_trial=20)


n_0_gam_1_ci <- boot.ci(CI_results, type="norm");n_0_gam_1_ci





## (3) MCMC-based method

require(rstan)

bern.stan ="

data {

  int<lower=0> N;              // Number of trials

  int<lower=0> K;              // Number of observations
```

```
  int<lower=0> y[N, K];      // Array of observed counts
}
parameters {
  real<lower=0, upper=1> theta[N]; // Probability vector for the Multinomial distribution
}
model {
  theta ~ beta(4, 6);  // Beta prior with parameters alpha = 1, beta = 1
  for (n in 1:N)
    y[n,] ~ bernoulli(theta[n]);    // Multinomial likelihood
}
"


nSim <- 1e4
y <- rbinom(n_trial, 1, P_SE[1])
for (i in 2:nSim) {
  y <- rbind(y,
        rbinom(n_trial, 1, P_SE[i]))
}
start_time <- Sys.time()
fit <-  stan(model_code=bern.stan,
        data=list(y=y, N=nSim, K =n_trial),
        #verbose = FALSE,
        iter=5000)
end_time <- Sys.time()
end_time - start_time
```

```
post_mean_list <- summary(fit)$summary[1:nSim,1]

## 4.331272 mins

## Time difference of 1.306908 hours


n_0_MCMC_1 <- n_trial * (var(P_SE) / var(post_mean_list) -
                 1);n_0_MCMC_1

## n_0 = 9.636626

## n_0(1e4) = 9.656048


## Bootstrat CI

n_0_dataset <- cbind(P_SE,post_mean_list)


CI_results <- boot(data=n_0_dataset, statistic=n_0_func,
           R=1000, n_trial=20)


n_0_MCMC_1_ci <- boot.ci(CI_results, type="norm");n_0_MCMC_1_ci




set.seed(3000615)

################## Experiment 2, Gamma-Exponential ##################

### 1. Obtain samples of theta from the prior distribution

nSim <- 1e5


n_0 <- 20 # n_0 is alpha parameter for the gamma prior distribution theta~gamma(a,b)
```

```
b <- 0.1 # beta parameter for the gamma distribution

## Draw sample
theta <- rgamma(nSim, shape = n_0, scale = b)
## Compute variance of theta
var.theta <- var(theta)
### 2. Obtain experimental data from the data likelihood in R
S <- numeric(nSim) # Initialize summary statistic S vector
n_trial.2 <- 100 # Additional data collections
## Generate data and compute summary statistic S

## (1) Variation ratio approach
## Compute variance of S
start_time <- Sys.time()
for (i in 1:nSim){
  k <- rexp(n_trial.2, theta[i])
  S[i] <- 1/mean(k)
}
var.S <- var(S)
## Estimate n0
n0.hat_2 <- n_trial.2*(var.S/var(theta)-1)
n0.hat_2
end_time <- Sys.time()
end_time - start_time

## Bootstrap CI
```

```
n_0_dataset <- cbind(theta,S)

CI_results <- boot(data=n_0_dataset, statistic=n_0_func_ss_med,
          R=1000, n_trial=n_trial.2)

n0.hat_2_ci <- boot.ci(CI_results, type="norm");n0.hat_2_ci



## (2) Nonparametric reg model approach

start_time <- Sys.time()
for (i in 1:nSim){
 k <- rexp(n_trial.2, theta[i])
 S[i] <- 1/mean(k)
}
post_X_gam_2 <- lm(theta ~ bSpline(1/S, degree = 4))

n_0_gam_2 <- n_trial.2 * (var(theta) / var(post_X_gam_2$fitted.values) -
             1);n_0_gam_2
end_time <- Sys.time()
end_time - start_time

## Bootstrap CI

n_0_dataset <- cbind(theta,post_X_gam_2$fitted.values)
```

```
CI_results <- boot(data=n_0_dataset, statistic=n_0_func,
          R=1000, n_trial=n_trial.2)

n_0_gam_2_ci <- boot.ci(CI_results, type="norm");n_0_gam_2_ci

## (3) MCMC-based method
require(rstan)
bern.stan ='
data {
  int<lower=0> N;      // Number of trials
  int<lower=0> K;      // Number of observations
  real<lower=0> y[N, K];   // Observed data (exponential random variable)
}

parameters {
  real<lower=0> theta[N];  // Probability parameter
}

model {
  theta ~ gamma(20, 1/0.1);  // Gamma prior with shape parameter alpha and rate
parameter beta
  for (n in 1:N)
    y[n,] ~ exponential(theta[n]); // Likelihood
}
'
```

```
nSim <- 1e4
y <-rexp(n_trial.2, theta[1])
for (j in 2:nSim) {
  y <- rbind(y,rexp(n_trial.2, theta[j]))
}
start_time <- Sys.time()
fit <-  stan(model_code=bern.stan,
        data=list(y=y, N=nSim, K =n_trial.2),
        #verbose = FALSE,
        iter=5000)
end_time <- Sys.time()
end_time - start_time
## 2.527916 mins
## Time difference of 49.97675 mins
post_mean_list <- summary(fit)$summary[1:nSim,1]

n_0_MCMC_2 <- n_trial.2 * (var(theta) / var(post_mean_list) -
              1);n_0_MCMC_2
## n_0 = 17.89942



## Bootstrap CI


n_0_dataset <- cbind(theta,post_mean_list)
```

```
CI_results <- boot(data=n_0_dataset, statistic=n_0_func,
          R=1000, n_trial=n_trial.2)

n_0_MCMC_2_ci <- boot.ci(CI_results, type="norm");n_0_MCMC_2_ci

set.seed(3000615)

################## Experiment 3, Poisson-Gamma ##################
### 1. Obtain samples of theta from the prior distribution
a <- 50 # alpha parameter for the gamma prior distribution theta~gamma(a,b)
n_0 <- 100 # n_0 is rate, or 1/beta parameter for the gamma distribution
nSim <- 1e5 # Number of samples from the prior
## Draw sample
theta <- rgamma(nSim, shape = a, rate = n_0)
## Compute variance of theta
var.theta <- var(theta)
### 2. Obtain experimental data from the data likelihood in R
S <- numeric(nSim) # Initialize summary statistic S vector
n_trial.3 <- 50 # Additional data collections


## (1) Variation ratio approach
## Compute variance of S
start_time <- Sys.time()
## Generate data and compute summary statistic S
```

```
for (i in 1:nSim){
  k <- rpois(n_trial.3, theta[i])
  S[i] <- mean(k)
}
var.S <- var(S)
## Estimate n0
n0.hat_3 <- n_trial.3*(var.S/var.theta-1)
n0.hat_3
end_time <- Sys.time()
end_time - start_time

## Bootstrap CI

n_0_dataset <- cbind(theta,S)

CI_results <- boot(data=n_0_dataset, statistic=n_0_func_ss_med,
            R=1000, n_trial=n_trial.3)

n0.hat_3_ci <- boot.ci(CI_results, type="norm");n0.hat_3_ci



## (2) Nonparametric reg model approach
## These two models have no difference
start_time <- Sys.time()
## Generate data and compute summary statistic S
for (i in 1:nSim){
```

```
  k <- rpois(n_trial.3, theta[i])
  S[i] <- mean(k)
}
post_X_gam_3 <- lm(theta ~ bSpline(S, degree = 6))

n_0_gam_3 <- n_trial.3 * (var(theta) / var(post_X_gam_3$fitted.values) -
                    1);n_0_gam_3
end_time <- Sys.time()
end_time - start_time

## Bootstrap CI

n_0_dataset <- cbind(theta,post_X_gam_3$fitted.values)

CI_results <- boot(data=n_0_dataset, statistic=n_0_func,
              R=1000, n_trial=n_trial.3)

n_0_gam_3_ci <- boot.ci(CI_results, type="norm");n_0_gam_3_ci

## (3) MCMC-based method
require(rstan)
bern.stan ='
data {
  int<lower=0> N;  // Number of trials simulation
  int<lower=0> K;      // Number of observations
  int<lower=0> y[N, K];  // Observed data (Poisson random variable)
```

```
}

parameters {
  real<lower=0> theta[N];  // Rate parameter
}

model {
  theta ~ gamma(50, 100);  // Gamma prior with shape parameter alpha and rate parameter
beta
  for (n in 1:N)
    y[n,] ~  poisson(theta[n]); // Likelihood
}
'

nSim <- 1e4
y <- rpois(n_trial.3, theta[1])
for (j in 2:nSim) {
  y <- rbind(y,rpois(n_trial.3, theta[j]))
}
start_time <- Sys.time()
fit <-  stan(model_code=bern.stan,
        data=list(y=y, N=nSim, K =n_trial.3),
        #verbose = FALSE,
        iter=5000)
end_time <- Sys.time()
end_time - start_time
```

```
## 10.17014 mins
post_mean_list <- summary(fit)$summary[1:nSim,1]

n_0_MCMC_3 <- n_trial.3 * (var(theta) / var(post_mean_list) -
                     1);n_0_MCMC_3
## n_0 = 98.91148



## Bootstrap CI

n_0_dataset <- cbind(theta,post_mean_list)

CI_results <- boot(data=n_0_dataset, statistic=n_0_func,
               R=1000, n_trial=n_trial.3)

n_0_MCMC_3_ci <- boot.ci(CI_results, type="norm");n_0_MCMC_3_ci

################## Experiment 4, Dirichlet-Multinomial ##################
### 1. Obtain samples of theta from the prior distribution
set.seed(3000615)
nSim <- 1e5
library(LaplacesDemon)
alpha_list <- c(10,5,8)
## True n_0 is 10+5+8 = 23
P_SE_dir <- rdirichlet(nSim, alpha_list)
```

```
### 2. Obtain experimental data from the data likelihood in R
S <- matrix(data = NA, nrow = nSim, ncol = length(alpha_list))


n_trial.4 <- 50 # Additional data collections


## (1) Variation ratio approach,
## each component produce the almost same n_0, and it should be!
## Compute variance of S for each component
start_time <- Sys.time()
## Generate data and compute summary statistic S
for (i in 1:nSim){
  k <- rmultinom(1,size = n_trial.4, P_SE_dir[i,])
  S[i,] <- k / n_trial.4
}


var.alpha1 <- var(P_SE_dir[,1])
## Estimate n0
n0.hat.1 <- n_trial.4*(var(S[,1])/ var.alpha1-1);n0.hat.1
end_time <- Sys.time()
end_time - start_time


## Compute variance of S for each component
var.alpha2 <- var(P_SE_dir[,2])
## Estimate n0
n0.hat.2 <- n_trial.4*(var(S[,2])/ var.alpha2-1);n0.hat.2
```

```
}

## Compute variance of S for each component
var.alpha3 <- var(P_SE_dir[,3])
## Estimate n0
n0.hat.3 <- n_trial.4*(var(S[,3])/ var.alpha3-1);n0.hat.3


## Bootstrap CI

n_0_dataset <- cbind(P_SE_dir[,1],S[,1])

CI_results <- boot(data=n_0_dataset, statistic=n_0_func_ss_med,
          R=1000, n_trial=n_trial.4)

n0.hat.1_4_ci <- boot.ci(CI_results, type="norm");n0.hat.1_4_ci



## (2) Nonparametric reg model approach
## These two models have no difference
start_time <- Sys.time()
## Generate data and compute summary statistic S
for (i in 1:nSim){
  k <- rmultinom(1,size = n_trial.4, P_SE_dir[i,])
  S[i,] <- k / n_trial.4
}
```

```
post_X_gam_4.1 <- lm(P_SE_dir[,1] ~ bSpline(S[,1], degree = 5))

n_0_gam_4.1 <- n_trial.4 * (var(P_SE_dir[,1]) / var(post_X_gam_4.1$fitted.values) -
                1);n_0_gam_4.1
end_time <- Sys.time()
end_time - start_time

# ## These two model should be same, S_2 S_3 provide no additional info for P_SE_dir[1]
# post_X_gam_4.1 <- lm(P_SE_dir[,1] ~ bSpline(S[,1], degree = 5)*
#             bSpline(S[,2], degree = 5)*
#             bSpline(S[,3], degree = 5))
#
# n_0_gam_4.1 <- n_trial.4 * (var(P_SE_dir[,1]) / var(post_X_gam_4.1$fitted.values) -
#                1);n_0_gam_4.1

post_X_gam_4.2 <- lm(P_SE_dir[,2] ~ bSpline(S[,2], degree = 5))

n_0_gam_4.2 <- n_trial.4 * (var(P_SE_dir[,2]) / var(post_X_gam_4.2$fitted.values) -
                1);n_0_gam_4.2

# ## These two model should be same, S_1 S_3 provide no additional info for P_SE_dir[2]
# post_X_gam_4.2 <- lm(P_SE_dir[,2] ~ bSpline(S[,1], degree = 5)*
#             bSpline(S[,2], degree = 5)*
#             bSpline(S[,3], degree = 5))
#
# n_0_gam_4.2 <- n_trial.4 * (var(P_SE_dir[,2]) / var(post_X_gam_4.2$fitted.values) -
```

```
#                         1);n_0_gam_4.2

post_X_gam_4.3 <- lm(P_SE_dir[,3] ~ bSpline(S[,3], degree = 5))

n_0_gam_4.3 <- n_trial.4 * (var(P_SE_dir[,3]) / var(post_X_gam_4.3$fitted.values) -
                        1);n_0_gam_4.3

## These two model should be same, S_1 S_3 provide no additional info for P_SE_dir[2]
# post_X_gam_4.3 <- lm(P_SE_dir[,3] ~ bSpline(S[,1], degree = 5)*
#                    bSpline(S[,2], degree = 5)*
#                    bSpline(S[,3], degree = 5))
#
# n_0_gam_4.3 <- n_trial.4 * (var(P_SE_dir[,3]) / var(post_X_gam_4.3$fitted.values) -
#                         1);n_0_gam_4.3

## Bootstrap CI

n_0_dataset <- cbind(P_SE_dir[,1],post_X_gam_4.1$fitted.values)

CI_results <- boot(data=n_0_dataset, statistic=n_0_func,
               R=1000, n_trial=n_trial.4)

n_0_gam_4.1_ci <- boot.ci(CI_results, type="norm");n_0_gam_4.1_ci
```

```
## (3) MCMC-based method

start_time <- Sys.time()

require(rstan)

bern.stan ="

data {

  int<lower=0> N;              // Number of trials

  int<lower=0> K;              // Number of categories

  int<lower=0> counts[N, K];     // Array of observed counts

  vector<lower=0>[K] alpha;      // Parameters of the Dirichlet prior

}


parameters {

  simplex[K] theta[N];            // Probability vector for the Multinomial distribution

}


model {

  theta ~ dirichlet(alpha);      // Dirichlet prior

  for (n in 1:N) {

    counts[n,] ~ multinomial(theta[n]);    // Multinomial likelihood

  }

}

"


nSim <- 1e4

post_mean_list <- rep(NA,nSim)

post_mean_df <- data.frame(matrix(NA,nSim,3))
```

```
y <- t(rmultinom(1,size = n_trial.4, P_SE_dir[1,]))
for (j in 2:nSim) {
  y <- rbind(y,t(rmultinom(1,size = n_trial.4, P_SE_dir[j,])))
}
data_list <- list(N = nSim, K = 3, counts = y, alpha=alpha_list)
start_time <- Sys.time()
fit <-  stan(model_code=bern.stan,
        data=data_list,
        #init = initial_values,
        chains = 4,
        #verbose = FALSE,
        iter=5000)
end_time <- Sys.time()
multi_time <- end_time - start_time
## Time difference: 15.05493 mins
n_0_MCMC_4.1 <- n_trial.4 * (var(P_SE_dir[,1]) /
               var(summary(fit)$summary[1:nSim*3-2,1]) -
               1);n_0_MCMC_4.1
## 22.67461
n_0_MCMC_4.2 <- n_trial.4 * (var(P_SE_dir[,2]) /
               var(summary(fit)$summary[1:nSim*3-1,1]) -
               1);n_0_MCMC_4.2
## 25.1234
n_0_MCMC_4.3 <- n_trial.4 * (var(P_SE_dir[,3]) /
               var(summary(fit)$summary[1:nSim*3,1]) -
```

```
                    1);n_0_MCMC_4.3
## 23.04029

## Bootstrap CI

n_0_dataset <- cbind(P_SE_dir[,1],summary(fit)$summary[1:nSim*3-2,1])

CI_results <- boot(data=n_0_dataset, statistic=n_0_func,
            R=1000, n_trial=n_trial.4)

n_0_MCMC_4.1_ci <- boot.ci(CI_results, type="norm");n_0_MCMC_4.1_ci

################## Experiment 5, Normal-Weibull ##################
### 1. Obtain samples of theta from the prior distribution in R
nSim <- 1e4
sigma_2 <- 0.04
mu_0 <- 1
n_0 <- NA # For noncongujate pair, n_0 is unknown

## Draw sample of theta
theta <- rnorm(nSim, mu_0, sd = sqrt(sigma_2))
## Compute variance of theta
var.theta <- var(theta)


## Generate Weibull data, take 6 mins
```

```
library(MASS)

weibull_data <- data.frame(matrix(NA,nSim,3))

colnames(weibull_data) <- c("theta","Post_mean","MLE_scale")

weibull_data[,1] <- theta




## (1) Variation ratio approach

## Compute variance of S

start_time <- Sys.time()

set.seed(3000614)

MLE.weibull.list <- rep(NA,nSim)

n_trial.5 <- 100

for (j in 1:nSim) {

  ### Number of patient included in the trial = 100

  y <- rweibull(n_trial.5, 1, 1/theta[j])

  fit.single.mle <- fitdistr(y, densfun = "weibull",

                        start = list(scale =1/theta[j]),

                        shape = 1,

                        method = "Nelder-Mead")

  MLE.weibull.list[j] <- 1/fit.single.mle$estimate

  print(cat("This is ", j, "iteration ", "\n"))

}

var.S <- var(MLE.weibull.list)

## Estimate n0

n0.hat_5 <- n_trial.5*( var.S/var.theta - 1)
```

```
n0.hat_5 ##   30.56851
end_time <- Sys.time()
end_time - start_time

## Bootstrap CI

n_0_dataset <- cbind(theta,MLE.weibull.list)

CI_results <- boot(data=n_0_dataset, statistic=n_0_func_ss_med,
          R=1000, n_trial=n_trial.5)

n0.hat.1_5_ci <- boot.ci(CI_results, type="norm");n0.hat.1_5_ci



## (2) Nonparametric reg model approach
## These two models have no difference
start_time <- Sys.time()
set.seed(3000613)
MLE.weibull.list <- rep(NA,nSim)
n_trial.5 <- 100
for (j in 1:nSim) {
 ### Number of patient included in the trial = 100
 y <- rweibull(n_trial.5, 1, 1/theta[j])
 fit.single.mle <- fitdistr(y, densfun = "weibull",
              start = list(scale =1/theta[j]),
```

```
                    shape = 1,
                    method = "Nelder-Mead")
  MLE.weibull.list[j] <- 1/fit.single.mle$estimate
  print(cat("This is ", j, "iteration ", "\n"))
}
post_X_gam_5 <- lm(theta ~ bSpline(1/MLE.weibull.list,
                        degree = 5))
n_0_gam_5 <- n_trial.5 * (var.theta / var(post_X_gam_5$fitted.values) -
                    1);n_0_gam_5 ## 24.94301
end_time <- Sys.time()
end_time - start_time

## Bootstrap CI

n_0_dataset <- cbind(theta,post_X_gam_5$fitted.values)

CI_results <- boot(data=n_0_dataset, statistic=n_0_func,
              R=1000, n_trial=n_trial.5)

n_0_gam_5.1_ci <- boot.ci(CI_results, type="norm");n_0_gam_5.1_ci



## (3) MCMC-based method
require(rstan)
bern.stan ="
data {
```

```
  int<lower=0> N;        // Number of Simulations
  int<lower=0> K;        // Number of observations in each simulation
  real<lower=0> y[N, K];  // Observed data (Weibull random variable)
}
parameters {
  real<lower=0> theta[N]; // chance of success
}
transformed parameters {
  real<lower=0> lambda[N]; // chance of success
  for (n in 1:N) {
    lambda[n] = 1/theta[n];       // prior
  }
}
model {
  for (n in 1:N) {
    theta[n] ~ normal(1, 0.2);       // prior
    y[n,] ~ weibull(1, lambda[n]);        // likelihood
  }
}
"

nSim <- 1e4
n_trial.5 <- 100
y <- t( rweibull(n_trial.5, 1, 1/theta[1]))
for (j in 2:nSim) {
  y <- rbind(y,t( rweibull(n_trial.5, 1, 1/theta[j])))
```

```
}
data_list <- list(N = nSim, K = n_trial.5, y = y)
start_time <- Sys.time()
set.seed(3000614)
fit <-  stan(model_code=bern.stan,
          data=data_list,
          chains = 4,
          #verbose = FALSE,
          iter=5000)
end_time <- Sys.time()
end_time - start_time
# Time difference of 9.263663 mins

n_0_MCMC_5 <- n_trial.5 * (var.theta /
                    var(summary(fit)$summary[1:nSim,1]) -
                    1);n_0_MCMC_5 ## 21.65507


## Bootstrap CI

n_0_dataset <- cbind(theta,summary(fit)$summary[1:nSim,1])

CI_results <- boot(data=n_0_dataset, statistic=n_0_func,
              R=1000, n_trial=n_trial.5)

n_0_MCMC_5_ci <- boot.ci(CI_results, type="norm");n_0_MCMC_5_ci
```

```
################## Experiment 6, Truncated normal-Binomial ##################

### 1. Obtain samples of theta from the prior distribution
set.seed(3000611)
library(truncnorm)
library(splines2)
nSim <- 1e4
P_SE <- rtruncnorm(nSim, a=0, b=1, mean = 0.2, sd = 0.1)
hist(P_SE)
### 2. Obtain experimental data from the data likelihood in R
n_trial <- 20
X_mean <- rbinom(nSim, n_trial, P_SE) / n_trial

## (1) Variation ratio approach
## Compute variance of S
start_time <- Sys.time()
y <- numeric(nSim) # Initialize summary statistic S vector
for (i in 1:nSim){
  y[i] <- rbinom(1, n_trial, P_SE[i])
}
S <- y / n_trial

var.S <- var(S)
## Estimate n0
```

```
n0.hat.6 <- n_trial*(var.S/var(P_SE)-1)
n0.hat.6
## 18.25749
end_time <- Sys.time()
end_time - start_time

## Bootstrap CI

n_0_dataset <- cbind(P_SE,S)

CI_results <- boot(data=n_0_dataset, statistic=n_0_func_ss_med,
          R=1000, n_trial=n_trial)

n0.hat.6_ci <- boot.ci(CI_results, type="norm");n0.hat.6_ci

## (2) Nonparametric reg model approach
start_time <- Sys.time()
X_mean <- rbinom(nSim, n_trial, P_SE) / n_trial
post_X_gam <- lm(P_SE ~ bSpline(X_mean, degree = 5))

n_0_gam_6 <- n_trial * (var(P_SE) / var(post_X_gam$fitted.values) -
             1);n_0_gam_6
## 16.66829
end_time <- Sys.time()
end_time - start_time
```

```
## Bootstrap CI

n_0_dataset <- cbind(P_SE,post_X_gam$fitted.values)

CI_results <- boot(data=n_0_dataset, statistic=n_0_func,
          R=1000, n_trial=n_trial)

n_0_gam_6_ci <- boot.ci(CI_results, type="norm");n_0_gam_6_ci

## (3) MCMC approach
stan_model_code <- "
functions {
 // Define the log probability of a truncated normal distribution
 real truncated_normal_lpdf(real x, real mu, real sigma, real lower, real upper) {
  real log_prob;
  log_prob = normal_lpdf(x | mu, sigma) -
   log_diff_exp(normal_lcdf(upper | mu, sigma),
          normal_lcdf(lower | mu, sigma));
  return log_prob;
 }
}

data {
 int<lower=0> N;     // Number of trials
 int<lower=0> K;        // Number of observations
 int<lower=0> y[N, K];   // Array of observed counts
```

```
}

parameters {
  real<lower=0, upper=1> p[N];  // Probability of success
}

model {
  for (n in 1:N){
    // Truncated normal prior for p
    target += truncated_normal_lpdf(p[n] | 0.2, 0.1, 0, 1);
    // Binomial likelihood
    y[n,] ~ bernoulli(p[n]);    // Multinomial likelihood
  }
}
"

library(rstan)

nSim <- 1e4
y <- rbinom(n_trial, 1, P_SE[1])
for (i in 2:nSim) {
  y <- rbind(y,
             rbinom(n_trial, 1, P_SE[i]))
}

# Assuming you have a vector of successes 'y' and a vector of attempts 'T'
```

```
# Prepare data for Stan model
start_time <- Sys.time()

stan_data <- list(
  N = nSim,
  y = y,
  K = n_trial
)
# Compile and fit the model
fit <- stan(
  model_code = stan_model_code,
  data = stan_data,
  iter = 5000,
  chains = 4
)

end_time <- Sys.time()
end_time - start_time
## 13.65727 mins
post_mean_list <- summary(fit)$summary[1:nSim,1]

n_0_MCMC.6 <- n_trial * (var(P_SE) / var(post_mean_list) -
                  1);n_0_MCMC.6

##16.19224
```

```
## Bootstrap CI

n_0_dataset <- cbind(P_SE,post_mean_list)

CI_results <- boot(data=n_0_dataset, statistic=n_0_func,
              R=1000, n_trial=n_trial)

n_0_MCMC.6_ci <- boot.ci(CI_results, type="norm");n_0_MCMC.6_ci

################## Experiment 7, Transformed Beta-Exponential ##################
### 1. Obtain samples of theta from the prior distribution in R
nSim <- 1e4
alpha <- 4
beta <- 6
n_0 <- alpha + beta

set.seed(3000614)
## Draw sample for parameters
P_SE_trans <- -log(1-rbeta(nSim, alpha, beta))



## Compute variance of theta
var.theta <- var(P_SE_trans)
### 2. Obtain experimental data from the data likelihood in R
## (1) Variation ratio approach
## Generate dataset
```

```
start_time <- Sys.time()

S <- numeric(nSim) # Initialize summary statistic S vector

n_trial.2 <- 100 # Additional data collections

## Generate data and compute summary statistic S

for (j in 1:nSim) {

  k <- rexp(n_trial.2, P_SE_trans[j])

  ## MLE of the exponential dist is the reciprocal of the sample mean!

  S[j] <- 1/mean(k)

}


var.S <- var(S)

## Estimate n0

## n_0 = 7.112813

##22.79408s


n0.hat.7 <- n_trial.2*(var.S/var(P_SE_trans)-1)

n0.hat.7

end_time <- Sys.time()

end_time - start_time

##* 7.403758


## Bootstrap CI


n_0_dataset <- cbind(P_SE_trans,S)


CI_results <- boot(data=n_0_dataset, statistic=n_0_func_ss_med,
```

```
            R=1000, n_trial=n_trial.2)

n0.hat.7_ci <- boot.ci(CI_results, type="norm");n0.hat.7_ci




## (2) Nonparametric reg model approach
## These two models have no difference
#post_X_gam_2 <- lm(theta ~ bSpline(S, degree = 5))
start_time <- Sys.time()
set.seed(3000614)
## Draw sample for parameters
P_SE_trans <- -log(1-rbeta(nSim, alpha, beta))
S <- numeric(nSim) # Initialize summary statistic S vector
n_trial.2 <- 100
for (j in 1:nSim) {
  k <- rexp(n_trial.2, P_SE_trans[j])
  ## MLE of the exponential dist is the reciprocal of the sample mean!
  S[j] <- 1/mean(k)
}
post_X_gam_2 <- lm(P_SE_trans ~ bSpline(S, degree = 5))

n_0_gam_2 <- n_trial.2 * (var(P_SE_trans) / var(post_X_gam_2$fitted.values) -
              1);n_0_gam_2
end_time <- Sys.time()
```

```
end_time - start_time

##* 4.87


## Bootstrap CI


n_0_dataset <- cbind(P_SE_trans,post_X_gam_2$fitted.values)


CI_results <- boot(data=n_0_dataset, statistic=n_0_func,
          R=1000, n_trial=n_trial.2)


n_0_gam_7_ci <- boot.ci(CI_results, type="norm");n_0_gam_7_ci


## (3) MCMC approach


stan_model_code <- "
data {
 int<lower=0> N;        // Number of observations
 int<lower=0> K;             // Number of observations
 real<lower=0> y[N, K];     // Array of observed counts
}


parameters {
 real<lower=0, upper=1> p[N]; // Parameter p following a Beta distribution
}


transformed parameters {
```

```
  vector<lower=0>[N] theta;       // Vector of transformed parameters


  for (n in 1:N) {
    theta[n] = -log1m(p[n]);      // Transform each p[n] to theta[n]
  }
}


model {
  for (n in 1:N){
    // Beta prior for p
    p[n] ~ beta(4, 6);


    // Loop through each observation for the exponential likelihood
    for (k in 1:K) {
      y[n, k] ~ exponential(theta[n]);
    }
  }
}
"
library(rstan)
nSim <- 1e4
alpha <- 4
beta <- 6
n_0 <- alpha + beta
P_SE_trans <- -log(1-rbeta(nSim, alpha, beta))
n_trial <- 100
```

```
set.seed(3000614)
y <-rexp(n_trial, P_SE_trans[1])
for (j in 2:nSim) {
  y <- rbind(y,rexp(n_trial, P_SE_trans[j]))
}

# Define your data
data_list_2 <- list(
  N = nSim,
  y = y,
  K = n_trial
)

# Fit the model
fit_7 <- stan(
  model_code = stan_model_code,  # Path to the Stan model file
  data = data_list_2,
  chains = 4,
  iter = 5000,
)

posterior_samples <- extract(fit_7)$theta

n_0_MCMC.7 <- n_trial * (var(P_SE_trans) / var(colMeans( posterior_samples ) )-
              1);n_0_MCMC.7
```

```
## n_0 = 4.90



## Bootstrap CI

n_0_dataset <- cbind(P_SE_trans,colMeans( posterior_samples ) )

CI_results <- boot(data=n_0_dataset, statistic=n_0_func,
           R=1000, n_trial=n_trial)

n_0_MCMC.7_ci <- boot.ci(CI_results, type="norm");n_0_MCMC.7_ci
```
